# Hydroxyethyl Starch 130/0.4 Binds to Neutrophils Impairing Their Chemotaxis through a Mac-1 Dependent Interaction

**DOI:** 10.3390/ijms20040817

**Published:** 2019-02-14

**Authors:** Alessandro Trentini, Francesca Murganti, Valentina Rosta, Carlo Cervellati, Maria Cristina Manfrinato, Savino Spadaro, Franco Dallocchio, Carlo Alberto Volta, Tiziana Bellini

**Affiliations:** 1 Section of Medical Biochemistry, Molecular Biology and Genetics, Department of Biomedical and Specialist Surgical Sciences, University of Ferrara, 44121 Ferrara, Italy; francesca.murganti@tu-dresden.de (F.M.); valentinarosta89@gmail.com (V.R.); crvcrl@unife.it (C.C.); mmc@unife.it (M.C.M.); dlf@unife.it (F.D.); blt@unife.it (T.B.); 2 Technische Universität Dresden, Research Center for Regenerative Therapies, 01307 Dresden, Germany; 3 Section of Anesthesia and Intensive Care, Department of Morphology, Surgery and Experimental Medicine, University of Ferrara, 44121 Ferrara, Italy; savinospadaro@gmail.com (S.S.); vlc@unife.it (C.A.V.)

**Keywords:** Hydroxyethyl Starch, Neutrophil, Chemotaxis, volume replacement solutions, fMLP, IL-8, integrin

## Abstract

Several studies showed that hydroxyethyl starch (HES), a synthetic colloid used in volume replacement therapies, interferes with leukocyte-endothelium interactions. Although still unclear, the mechanism seems to involve the inhibition of neutrophils’ integrin. With the aim to provide direct evidence of the binding of HES to neutrophils and to investigate the influence of HES on neutrophil chemotaxis, we isolated and treated the cells with different concentrations of fluorescein-conjugated HES (HES-FITC), with or without different stimuli (N-Formylmethionine-leucyl-phenylalanine, fMLP, or IL-8). HES internalization was evaluated by trypan blue quenching and ammonium chloride treatment. Chemotaxis was evaluated by under-agarose assay after pretreatment of the cells with HES or a balanced saline solution. The integrin interacting with HES was identified by using specific blocking antibodies. Our results showed that HES-FITC binds to the plasma membrane of neutrophils without being internalized. Additionally, the cell-associated fluorescence increased after stimulation of neutrophils with fMLP (*p* < 0.01) but not IL-8. HES treatment impaired the chemotaxis only towards fMLP, event mainly ascribed to the inhibition of CD-11b (Mac-1 integrin) activity. Therefore, the observed effect mediated by HES should be taken into account during volume replacement therapies. Thus, HES treatment could be advantageous in clinical conditions where a low activation/recruitment of neutrophils may be beneficial, but may be harmful when unimpaired immune functions are mandatory.

## 1. Introduction

Fluid resuscitation with colloid and crystalloid solutions is one of the main interventions in the management of critically ill patients. Although the main role of volume replacement solutions lie in the restoration of intravascular volume, they have also proved to be potent modulators of inflammation at both the systemic and cellular levels, demonstrating either anti-inflammatory or pro-inflammatory effects [1,2]. Among the semisynthetic colloids, one of the most commonly used worldwide is hydroxyethyl starch (HES), which is produced by hydroxyethyl substitution of amylopectin. HES has shown an increased persistence in the intravascular space compared to other colloids [3] and has been demonstrated to possess anti-inflammatory properties [4,5]. For instance, a wealth of evidence indicates that HES is able to modulate several leukocyte functions such as degranulation [6], oxidative burst [7,8,9,10] and neutrophil–endothelium interaction [11]. This last aspect is of particular relevance, considering the role of leukocytes in inducing microvascular permeability and endothelial damage [12,13]. Indeed, a large body of evidence suggests that neutrophil adhesion to the endothelium and their extravasation during inflammatory conditions might contribute to vascular leakage, either directly or through a paracrine pathway sustained by the release of granules [12]. Thus, the control of the adhesion of neutrophils to endothelium might be beneficial in reducing organ dysfunction and vascular leakage [4,14]. On the other hand, a strong inhibition of adhesion in conditions where the role of neutrophils may be crucial, e.g. during infections, could worsen patient outcome [15]. Previous studies showed that HES was able to impair leukocytes–endothelial interactions and neutrophil trans-endothelial migration. However, there are still conflicting results regarding the mechanisms by which HES can affect the leukocyte–endothelium couple, although a supposed inhibitory effect on neutrophil integrins had been advocated [16,17]. To address this question, in the present study we assessed the binding of HES to neutrophils and the effect of specific integrin-blocking antibodies on this interaction. Moreover, we evaluated the influence of HES on the chemotaxis of neutrophils in response to IL-8 and fMLP, in order to estimate the impact of HES-containing volume replacement solutions on neutrophils’ activity during in vitro simulated inflammatory conditions.

## 2. Results

### 2.1. Hydroxyethyl Starch (HES) Binds to the External Plasma Membrane of Neutrophils

**Figure 1:**
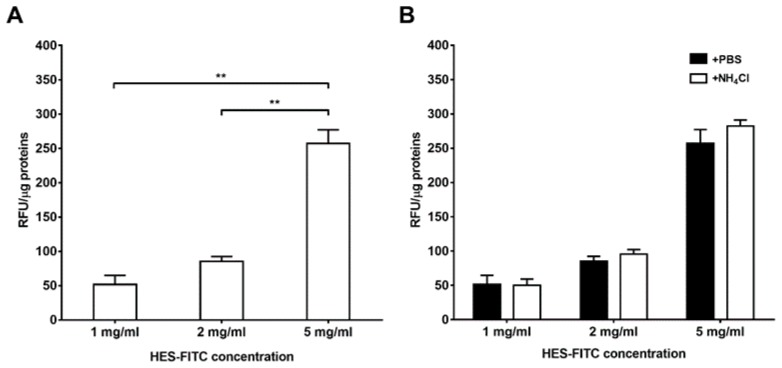
Association of HES to the outer plasma membrane of neutrophils. (**A**) Neutrophils were treated with different concentrations of FITC-labeled HES, washed and the resulting fluorescence read with a microplate fluorimeter. There was an increase in fluorescence with increasing concentrations of HES-FITC (*n* = 3). (**B**) After the treatment with HES-FITC and washing steps, neutrophils were incubated with PBS or NH_4_Cl in order to rule out a possible internalization of HES into phagolysosomes. No significant difference in the fluorescence of the cells treated with NH_4_Cl compared to the control was observed, suggesting that HES was bound to the outer plasma membrane and not internalized (*n* = 3). The data are presented as mean ± SD ** *p* < 0.01.

To evaluate the binding of HES to neutrophils, cells were treated with different concentrations of HES labeled with fluorescein isothiocyanate (FITC) and the fluorescence intensity was read with a microplate reader. As depicted in Figure 1A, the maximum binding of HES to neutrophils was detected at 5 mg/mL, as observed by the mean fluorescence at each HES-FITC concentration adjusted for the total proteins present in the wells (Figure 1A, *p* < 0.01 with respect to both 1 mg/mL and 2 mg/mL). Since HES can be synthesized starting from different raw materials (e.g. maize or potato), with various molar substitution and C2/C6 ratios [18], we further tested whether HES from these two sources showed the same binding affinity for neutrophils. The two kinds of HES substantially showed the same binding effect, suggesting a sort of bioequivalence for the two starches with respect to binding to neutrophils (Figure S1).

In order to rule out a possible internalization of HES by phagocytosis or other processes, neutrophils were treated with ammonium chloride, a lysosomotropic agent that increases the intracellular pH resulting in an enhancement of the FITC fluorescence intensity. No significant difference in the fluorescence intensity after the treatment of the cells with ammonium chloride compared to the control at any tested concentration of HES-FITC was observed (Figure 1B). Together, these findings suggested that HES was able to bind to the outer plasma membrane without being internalized.

Finally, to confirm the association of HES to the plasma membrane of neutrophils, the cells were incubated with different concentrations of HES-FITC and the fluorescence intensity was measured under two different conditions: at pH 5.8, similar to the intravacuolar pH, and at pH 5.8 after the treatment of cells with trypan blue, a quencher of the extracellular fluorescence. After the treatment with trypan blue, a decreased fluorescence intensity at each concentration of HES compared to the control was observed, with a mean quenching of the signal of about 97 ± 2%, confirming the binding of HES to the external plasma membrane (Table 1).

### 2.2. The Binding of HES to Neutrophils Increased after Stimulation

**Figure 2:**
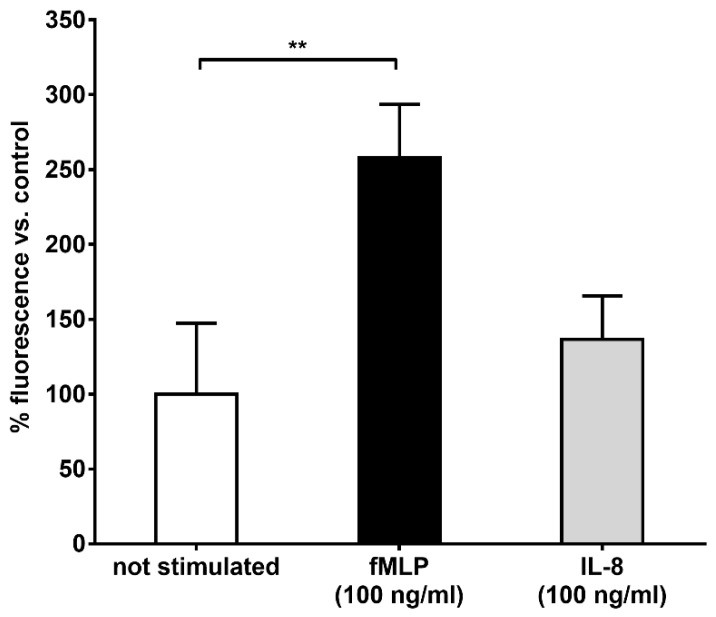
Increase in the binding of hydroxyethyl starch (HES) after neutrophils stimulation. Neutrophils were either activated with fMLP, IL-8 or not stimulated and then incubated with HES-FITC. After washing steps, the fluorescence was read with a microplate fluorimeter and the values were reported as percentage of binding with respect to the not stimulated condition. There was a significant increase in the binding of HES after fMLP stimulation but not after IL-8 treatment. The data represents the mean ± SD of five independent experiments. ** *p* < 0.01.

Neutrophils isolated from fresh buffy coats were fully responsive to stimulation (as outlined in Figure S2). To determine whether the binding of HES to the plasma membrane could be influenced by different stimuli, the cells were treated with either fMLP, IL-8 or not stimulated in the presence of HES-FITC and the resulting fluorescence measured. We observed an increase in the binding of HES after treatment of neutrophils with fMLP compared to the control (Figure 2). In contrast, no significant difference in the fluorescence after stimulation with IL-8 was detected (Figure 2).

### 2.3. Hydroxyethyl Starch Impairs the Chemotaxis of Neutrophils

**Figure 3:**
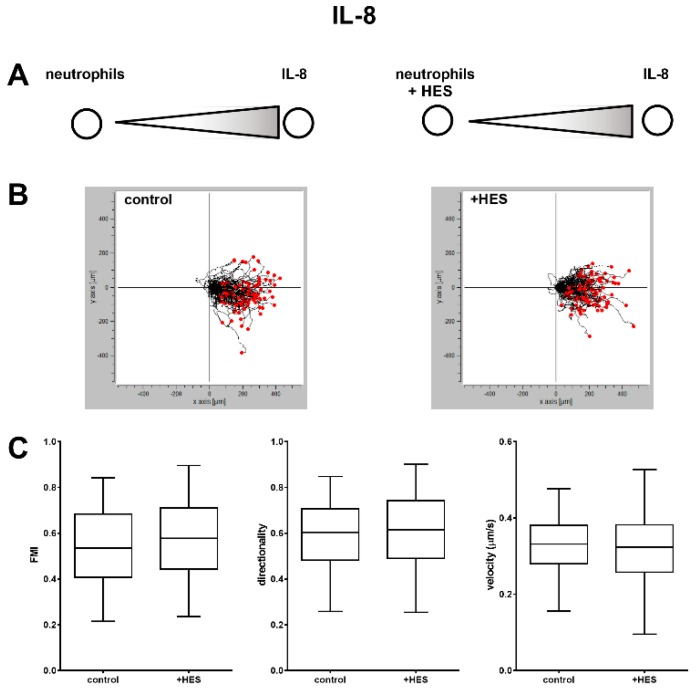
Migration of neutrophils not treated (control) or treated (+ HES) with hydroxyethyl starch in response to IL-8. Low magnification time-lapse images were obtained and processed using cell-tracking algorithms. (**A**) Experimental setup. (**B**) Detailed migration trajectories of representative cells (*n* = 100) from control and HES-treated cells; individual tracks were transposed so that each had its start at the origin. (**C**) Comparison of forward migration index (FMI), parallel to the gradient and calculated as the ratio between the x coordinate of the cell’s end point of migration and the total distance accumulated), directionality (representing a measurement of the directness of cell trajectories, the ratio of the Euclidian distance and the total accumulated distance of a cell), and velocity (displacement/time). None of the values were different between the control and the cells treated with HES, suggesting that hydroxyethyl starch did not impair the migration of cells in response to IL-8. The results are the mean ± SD of three independent experiments.

**Figure 4:**
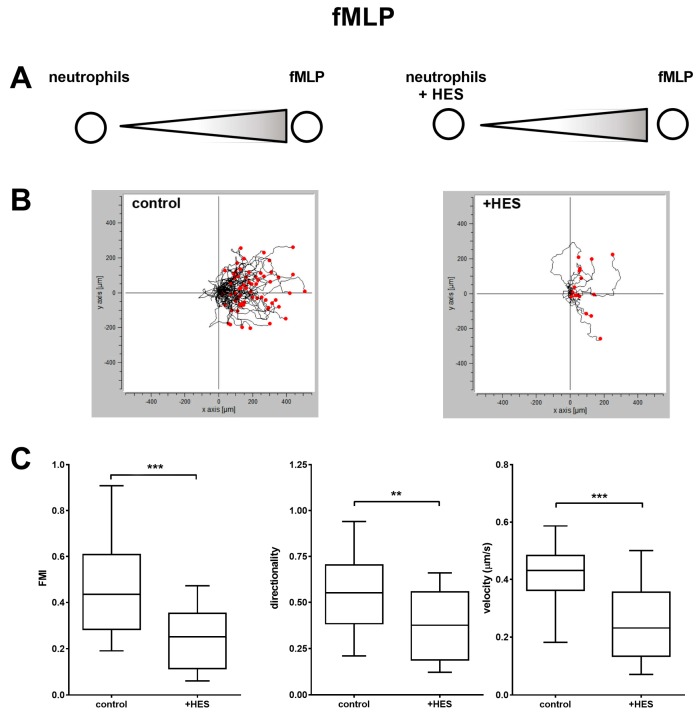
Migration of neutrophils not treated (control) or treated (+ HES) with Hydroxyethyl starch in response to fMLP. Low magnification time-lapse images were obtained and processed using cell-tracking algorithms. (**A**) Experimental setup. (**B**) Detailed migration trajectories of representative cells from control (*n* = 62) and HES-treated (*n* = 17) cells; individual tracks were transposed so that each had its start at the origin. (**C**) Comparison of forward migration index, directionality, and velocity. As can be seen, the cells pre-treated with HES showed a decreased FMI, directionality and velocity compared to the control. The results are the mean ± SD of three independent experiments. ** *p* < 0.01, *** *p* < 0.001

Based on the above findings, cells were pretreated with HES or not treated as a control and then subjected to an under-agarose migration assay towards IL-8 (Figure 3A) or fMLP (Figure 4A). The migration paths in response to IL-8 were not substantially different between the control and the cells treated with HES (Figure 3B). In agreement with this, we did not observe any statistical difference in FMI (forward migration index), directionality and velocity between the HES-treated and untreated cells (Figure 3C), suggesting that HES did not impair the migration of neutrophils towards IL-8.

On the contrary, when cells were pretreated with HES and subjected to the migration assay with fMLP as a chemoattractant, we observed more convoluted tracks for the HES-treated cells (Figure 4B) and a significant decrease in both FMI, directionality and velocity compared to the control (Figure 4C). Of note, we observed a strong decrease in the cell density after treatment with HES only for the migration towards fMLP but not IL-8 (data not shown).

### 2.4. The Binding of HES to Neutrophils is Mediated by Mac-1 Activating the Outside-in Signaling of the Integrin

**Figure 5:**
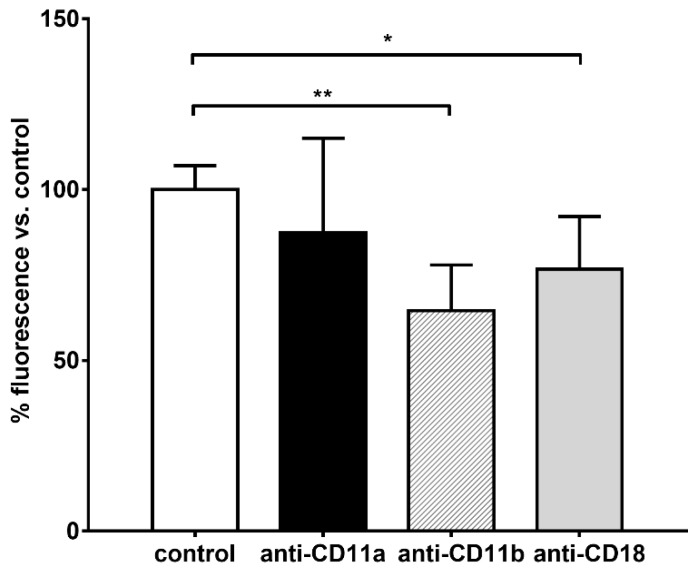
Blocking of integrin subunits with specific antibodies. The cells were incubated with blocking antibodies specific for either CD11a (black bar), CD11b (dashed bar), CD18 (gray bar) or isotypic control (open bar) and treated with HES-FITC. The fluorescence was recorded after washing steps and the data were represented as percentage of fluorescence with respect to the control. We observed that the blocking of CD11b or CD18 subunits, but not CD11a, prevented the binding of HES (decrease in the membrane-associated fluorescence) to neutrophils. The data represent the mean ± SD of five independent experiments. * *p* < 0.05; ** *p* < 0.01.

The main integrins in neutrophils that drives the migration towards chemo-attractants are LFA-1 (composed of CD11a and CD18 subunits) and Mac-1 (composed of CD11b and CD18 subunits). While LFA-1 is mainly used for the migration in response to IL8, Mac-1 drives the movement of the cells towards fMLP [19]. To confirm that the binding of HES to the plasma membrane of neutrophils was mainly due to Mac-1, neutrophils were treated with specific blocking antibodies against CD11a (LFA-1), CD11b (Mac-1) or the common subunit CD18, then exposed to FITC-labeled HES. We observed a significant decrease in the fluorescence of the cells of about 46% and 37% with respect to the control after treatment with anti-CD11b and anti-CD18 blocking antibodies, respectively (Figure 5).

Moreover, we did not find any significant difference (*p* = 0.7822) between the control and the cells treated with anti-CD11a antibodies, suggesting that the HES might not be a ligand of the CD11a integrin subunit.

**Figure 6:**
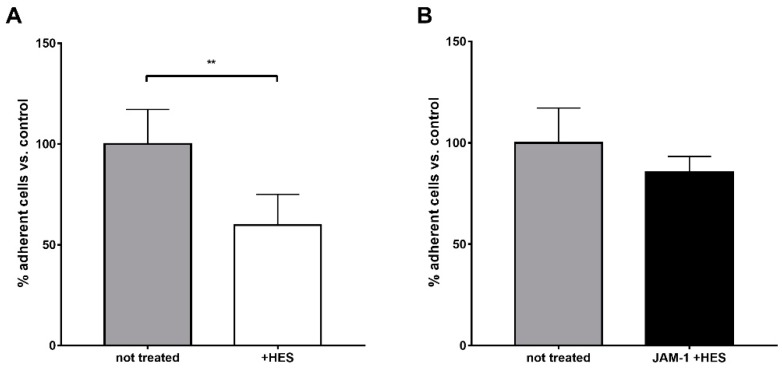
Static adhesion assay performed on different substrates. Cells were fluorescently labeled with calcein and then subjected to adhesion on different substrates. (**A**) When fibrinogen was used as a substrate for adhesion, HES pre-treatment strikingly decreased (almost 50%) the adhesion of cells. (**B**) The adhesion of neutrophils on JAM-1 was not impaired by HES, suggesting a Mac-1 specific effect. The presented results are the mean ± SD of four independent experiments. ** *p* < 0.01.

These results were also confirmed by a static adhesion assay on different substrates, fibrinogen and Junction Adhesion Molecule-1 (JAM-1): while fibrinogen is a specific substrate for Mac-1 [20], JAM-1 is preferentially bound by the LFA-1 integrin [21]. A decrease of almost 50% in the neutrophils’ adhesion on fibrinogen was observed upon treatment with HES (Figure 6A), whereas HES did not significantly affect the adhesion of the cells on JAM-1 (Figure 6B). Together, these findings confirmed the specific impairment of Mac-1-dependent adhesion. 

**Figure 7:**
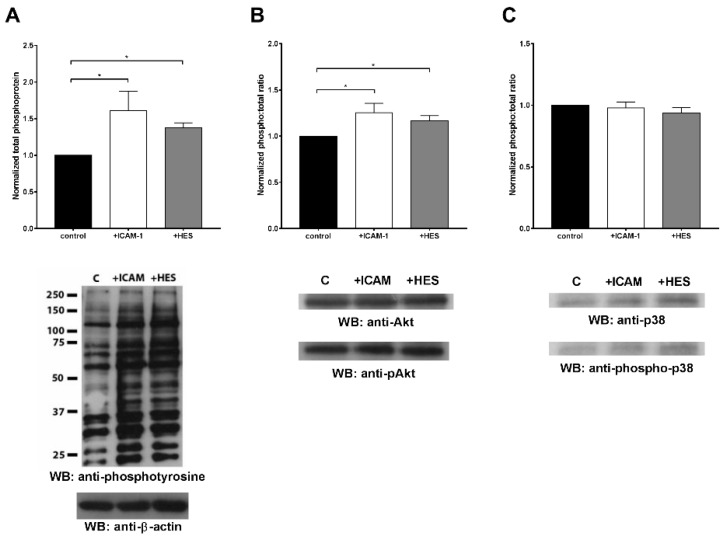
Evaluation of integrin activation by HES. (**A**) HES-mediated outside-in integrin activation was evaluated by determining total phosphorylation of tyrosine on intracellular proteins through Western blot (only a representative blot is showed in the picture). HES caused a significant increase in total phosphorylation compared to the control. (**B**) Evaluation of Akt activation by Western blot. HES treatment significantly increased the phosphorylation of Akt the activation of this pathway. (**C**) Evaluation of p38 activation by Western blot. As reported, neither HES nor ICAM caused the activation of the p38/MAPK pathway. In the blots, lane C represents the control. The presented results are the mean ± SD of three independent experiments. * *p* < 0.05.

Finally, we determined whether HES behaves as an integrin ligand, thus inducing its activation and initiating outside-in signaling. For this purpose, we analyzed the overall intracellular protein tyrosine phosphorylation in neutrophils treated with ICAM-1, HES or without any treatment (Figure 7A). HES broadly stimulated protein tyrosine phosphorylation to an extent similar to ICAM-1 (Figure 7A, top).

In addition, we evaluated the activation of two possible Mac-1 outside-in signaling pathways: PI3K/Akt and p38/MAPK pathways. We found that Mac-1 activation by HES induced PI3K/Akt signaling as determined by the increase in phosphorylated Akt (Figure 7B). On the contrary, p38 was not activated by HES (Figure 7C). Of note, as reported in other studies [22], ICAM-1 activated PI3K/Akt but not p38/MAPK signaling. 

Together these findings suggest the ability of HES to induce integrin activation and outside-in signaling, therefore behaving like a “physiological” ligand.

## 3. Discussion

The interaction between endothelial cells and neutrophils has a pivotal role in the early stages of acute inflammation. Thanks to this interaction, neutrophils can migrate towards the site of injury to eradicate noxious agents. However, when this process is massive and uncontrolled it can increase the already present vascular permeability, exacerbating vascular leak syndrome [12,23], a condition found in several critically ill patients. Thus, the control of neutrophil adhesion to the endothelium and of trans-endothelial migration may have large impact on clinical practice.

Previous studies showed that HES is able to impair the neutrophil–endothelial cell interaction. This occurs probably by inhibiting neutrophil integrin function, revealed by a decrease in firm adhesion and trans-endothelial migration without affecting rolling efficiency [16,17]. On the other hand, other studies suggested that HES decreased neutrophil adhesion through an endothelial-dependent mechanism, since only the treatment of endothelial cells with HES was found to affect adhesion of leukocytes, leuko-aggregation and rolling velocity [11,24], along with decreased endothelial activation [25]. Thus, currently published data on the topic are still highly conflicting and, overall, lack direct evidence of the binding of HES to neutrophils, which is supportive of any of the aforementioned mechanistic hypotheses. 

Owing these premises, our study aimed to evaluate in vitro the physical interaction between HES and neutrophils, and to investigate the influence of HES on neutrophil chemotaxis towards different stimuli as well as the players involved in HES binding. As highlighted by our results, for the first time we were able to directly demonstrate that HES is able to solely bind to the extracellular side of the plasma membrane without being internalized. Indeed, by using a fluorescently labelled HES (HES-FITC) we observed increasing cell-associated fluorescence with increasing HES-FITC concentration, fluorescence that was completely abolished upon treatment with a quenching agent. In addition, treatment with ammonium chloride, a lysosomotropic agent, did not exert any effect on FITC fluorescence, therefore confirming that HES is not internalized into phagosomes. 

It is commonly accepted that integrins exist on circulating cells in a dynamic equilibrium between three states with different ligand affinities: low, intermediate, and high [22]. Several studies reported that the ligation of integrins with substrates shifts this equilibrium towards the high-affinity state starting the outside-in signaling cascade, leading to phosphorylation of intracellular proteins and various effects. Therefore, from our data we can hypothesize that HES may behave as a physiological substrate for integrins by shifting the equilibrium towards the high-affinity state, further demonstrating its ability to bind to resting cells. Moreover, this binding seems sufficient to trigger outside-in signaling, as evidenced by the increased total phosphorylation of tyrosine found in intracellular proteins, events that can be further translated into the activation of signaling cascades modulating neutrophil responses. Indeed, from our results we observed that HES is able to activate the PI3K/Akt pathway, leaving p38/MAPK unaffected. The activation of Akt was previously associated with the modulation of lifespan [22] and oxidative burst [26] of neutrophils. However, the specific effect of HES on the activation of these cells is still a matter of debate and inconclusive. Therefore, our results suggested that the beneficial effect of HES observed in other studies may be at least partially dependent on neutrophil-associated mechanisms [16,17]. This is also in line with recent observations from Rossaint et al. on an animal model where HES was able to decrease inflammation, neutrophil recruitment in several organs, and their activation as measured by extracellular trap formation [27]. Nonetheless, we cannot completely exclude that the anti-inflammatory effects of HES [27,28] may be mediated by mechanisms also involving endothelial cells. In fact, it has been shown that HES can be internalized through pinocytosis by HUVEC (Human Umbilical Vein Endothelial Cell) [29], although no firm binding of the starch on the surface of these cells has been observed. Furthermore, a decreased expression of adhesion molecules, in particular E-selectin, on endothelial cells after treatment with HES has been reported [11,30]. However, contrasting findings have been also reported [16,17,29]. 

Differently, the body of literature dealing with the interaction between HES and neutrophils is highly consistent and points to the significant impact of synthetic colloids on the functionality of these immune cells. Besides the previous interference on degranulation and bacteria killing capacity [6,8], our results confirmed that chemotaxis is also massively impaired, with a striking decrease in cell migration (FMI, velocity and directionality) only towards “end-target” chemoattractants (e.g. fMLP) but not IL-8. As revealed by our results, the decrease in both FMI and velocity are mainly due to a disruption of Mac-1 (CD11b/CD18) action. Indeed, we observed that blocking the CD11b and, to a lesser extent, the CD18 subunits with specific antibodies was able to prevent the binding of HES-FITC to the cell surface with a supposed cooperative mechanism, since the cumulative percentage decrease in fluorescence intensity was almost 80%, whereas blocking the CD11a subunit did not exert any effect. This result is also supported by the finding of a significant increase in fluorescence intensity after treatment with fMLP but not IL-8, stimuli that can differently affect the affinity of the two integrins for their ligands with an increased avidity of Mac-1 following fMLP stimulation [31]. Moreover, this was further confirmed by the finding of an impaired adhesion on fibrinogen, a CD11b specific substrate [20], without affecting adhesion to JAM-1, a preferential substrate of CD11a [21]. Of note, the impairment in directionality we observed in cell migration towards fMLP after treatment with HES could be due to the interaction of HES with alpha 4-integrins as well. Indeed, it is known that directionality in neutrophil chemotaxis is mediated by the action of alpha-4 and LFA-1 integrins [32]; since we did not observe significant binding of HES to LFA-1, the only possible interactor remains alpha-4 integrin. However, without further experiments this remains mere speculation.

Collectively, our results might help to explain previous data showing an alteration in the rate of trans-endothelial migration of neutrophils treated with HES without impairing their rolling on activated endothelial cells. Indeed, while rolling is mainly mediated by selectins [33], the two integrins expressed on neutrophils’ surfaces serve different functions during the migration cascade: on one side, LFA-1 seems to be more involved in early adhesion strengthening to the endothelium [34]; on the other side, Mac-1 is essential for the intraluminal crawling of neutrophils to emigration sites [35]. Thus, by preventing the binding of Mac-1 to its ligand, HES may be able to alter intraluminal crawling, controlling neutrophil migration to sites of inflammation. However, the observed lack of binding of HES to LFA-1 disagreed with the decreased tethering rate from other studies [16,17], although this might be due to differences in the employed techniques. In fact, only flow-chamber assays can be used to study neutrophil arrest and trans-endothelial migration in vitro, whereas the assays we used do not mimic dynamic conditions such as blood flow.

Nonetheless, the specific effect we observed may have a large impact on clinical practice, especially when a low activation of neutrophils may be advantageous, such as during sterile inflammation [33] and ischemia/reperfusion injury [36,37], where the blocking of integrin activation has proven to be effective in preventing tissue damage [38]. On the other hand, when perfect functionality of neutrophils are mandatory, such as in the context of bacterial inflammation, the perturbation of the adhesion cascade may be more harmful than beneficial [27].

This study was not without limitations. First, the lack of data on the affinity of Mac-1 and LFA-1 for HES in an isolated system and different activation states of integrins. Second, the use of the “static” under agarose assay instead of a flow-chamber assay may have weakened our data, although this is the first study identifying Mac-1 as the interacting partner of HES. Third, we did not perform experiments on activated endothelial cells to confirm/deny that HES firmly binds to these cells.

In conclusion, the binding of HES to the extracellular side of the plasma membrane of neutrophils and the consequent impairment of chemotaxis can be pictured as an anti-inflammatory property of the molecule. This is of particular interest, considering the long persistence of HES in the extravascular space. Thus, the observed effect can be largely dependent on its vascular elimination and may have an impact on the choice of volume replacement solution in clinical settings. Therefore, the type of inflammatory condition of patients should be an additional parameter to consider in the choice of the volume replacement strategy in order to exploit the possible immunomodulatory properties of hydroxyethyl starch. Further studies are necessary to determine the real clinical relevance of our findings.

## 4. Materials and Methods

### 4.1. Reagents

Ficoll-Hypaque Plus was purchased from GE Healthcare (Milan, Italy; Cat. No. 17-1440-02). Dextran 500 (Cat. No. 31392), FITC isomer I (Cat. No. F7250), ethanol absolute (Cat. No. 02860), fMLP (Cat. No. F3506), agarose (Cat. No. A9045), RPMI-1640 (Cat. No. R6504), bovine serum albumin (BSA, Cat. No. A8022), bicinchoninic acid assay kit (Cat. No. BCA1) were purchased from Sigma-Aldrich (Milan, Italy). HES (Tetraspan) was obtained from BBraun (Melsungen, Germany). Fetal bovine serum (FBS) was purchased from Immunological Sciences (Rome, Italy; Cat. No. EU-000-500). DMSO (dimethyl sulfoxide) was purchased from Merck (Darmstadt, Germany; Cat. No. 1.09678.0100). IL-8 was purchased from Peptrotech (London, UK; Cat. No. 200-08M). Anti-CD11a (LFA-1; Cat. No. an3981), anti-CD11b (MAC-1; Cat. No. ab130428), and anti-CD18 (Cat. No. ab8220) blocking antibodies were purchased from Abcam (Cambridge, UK) and isotypic antibodies were from Santa Cruz Biotechnology (Dallas, TX, USA). Anti pAkt1/2/3 (Cat. No. sc515451), total Akt1/2/3 (Cat. No. sc81434), phosphorylated p38 (Cat. No. sc166182) and total p38 (Cat. No. sc33688) were from Santa Cruz Biotechnology (Dallas, TX, USA).

### 4.2. Labeling of HES with Fluorescein Isothiocyanate (FITC)

The labeling of HES with FITC was carried out according to the method proposed by Ständer and coworkers [39], with some modifications. Briefly, 0.1 g of HES was dissolved in 1.2 mL of DMSO and heated at 95 °C. After the complete dissolution of HES, 10 mg of FITC were added to the mixture and incubated for 6 h at 95 °C. The solution was cooled to room temperature and was added to 8 mL of absolute ethanol to precipitate the labeled HES. After a centrifugation of 10 min at 3300 rpm the supernatant was discarded and the pellet suspended in 1.6 mL of distilled water and dialyzed extensively against water. The final isolation of the labeled HES was done by freeze drying.

### 4.3. Isolation of Neutrophils and Evaluation of the Binding of HES to Their Plasma Membrane

Buffy coats were collected from the Blood Bank of S. Anna Hospital, Ferrara. All the data were analyzed anonymously, and the authors did not have any sensitive information about the participants. Neutrophils were isolated by gradient centrifugation and dextran sedimentation as previously described [6]. The cells were further suspended in HBSS (5.3 mM KCl, 0.44 mM KH_2_PO_4_, 138 mM NaCl, 0.3 mM Na_2_HPO_4_, 10 mM HEPES, 5.6 mM Glucose, pH 7.4) without calcium and magnesium and kept on ice until use. 

In order to evaluate the binding of HES to the plasma membrane of neutrophils, the cells were first treated with different concentrations of HES-FITC and then, after washing steps, the cell-associated fluorescence intensity was measured. Cells were further treated with ammonium chloride (NH_4_Cl), a lysosomotropic agent that penetrate in the cells raising the pH of the phagolysosome [40] thus enhancing the fluorescence of the FITC. Finally, to confirm the association of HES to the outer plasma membrane, the cells were treated with trypan blue in order to quench the extracellular fluorochrome emission [40].

Briefly, freshly-isolated neutrophils (8 × 10^6^ cells) were suspended in HBSS containing different concentrations of HES-FITC (1 mg/mL, 2 mg/mL or 5 mg/mL) dissolved in HBSS with 1 mM of CaCl_2_ and MgCl_2_, (herein referred to as complete HBSS) and incubated for 15 min at 37 °C. The highest concentration of HES (5 mg/mL) resembles the plasma concentration observed within the first 6 h after a bolus infusion of 500–1000 mL of the starch [16]. After incubation, cells were centrifuged for 10 min at 1100 rpm at 4 °C and washed twice with PBS. Finally, the pellets were suspended in 1 mL of complete HBSS and 100 µL of suspension (800,000 cells) were dispensed in quadruplicate in a clear flat bottomed 96 wells microtiter plate (Greiner Bio-One, Cat. No. 655101) and the fluorescence of FITC was read with a microplate fluorimeter (Tecan Infinite M200, Austria) at λ_ecc_ = 490 nm and λ_em_ = 520 nm. After the reading, 50 µL of PBS were added into two wells and 50 µL of 150 mM ammonium chloride (NH_4_Cl, 50 mM final concentration) were added to the remaining wells and the fluorescence was measured again.

To quench the signal coming from the plasma membrane, neutrophils (8 × 10^6^ cells) were suspended in 1 ml of acetate buffer (20 mM acetate buffer, pH 5.8 containing 130 mM NaCl) and 100 µL of suspension (800,000 cells) were dispensed in quadruplicate in a clear flat bottomed 96 wells microtiter plate and the fluorescence of FITC read with a microplate fluorimeter. The remaining cells were incubated with Trypan Blue dissolved in acetate buffer at a final concentration of 0.2 mg/mL for 20 s at room temperature. Cells were then washed twice with acetate buffer and suspended in the same buffer. Finally, 100 µL of suspension (800,000 cells) were dispensed in quadruplicate and the resulting fluorescence read with a microplate fluorimeter.

### 4.4. Binding of HES to the Plasma Membrane of Stimulated Neutrophils

Neutrophils (8 × 10^6^ cells) were suspended in complete HBSS containing 5 mg/mL HES-FITC and 100 ng/mL fMLP, 100 ng/mL IL-8 or without stimulation and incubated for 15 min at 37 °C. At the end of the incubation, the neutrophils were centrifuged at 1100 rpm at 4 °C for 10 min and washed twice with PBS. The cells were then suspended in 1 mL of complete HBSS and 100 µL of suspension (800,000 cells) were dispensed in quadruplicate in a clear flat bottomed 96 wells microtiter plate, and the fluorescence of FITC was read with a microplate fluorimeter.

### 4.5. Binding of HES after Treatment of Neutrophils with Integrin-Blocking Antibodies

Neutrophils (1 × 10^6^ cells) were suspended in complete HBSS and incubated for 30 min at room temperature with 20 µg/mL (corresponding to 2 µg of antibodies per million of cells) of anti-CD11a (LFA-1, Abcam, Cat. No. AB3981, clone MEM-83), anti-CD11b (Mac-1, Abcam, Cat. No. AB130428, clone ICRF44), anti-CD18 (Abcam, Cat. No. AB8220, clone MEM-148) blocking antibodies, or with isotypic antibodies as a control. Afterwards, the cells were centrifuged at 1100 rpm for 5 min, treated with 5 mg/mL HES-FITC dissolved in complete HBSS. Cells were then incubated for 15 min at room temperature in the dark. Finally, the cells were washed twice with PBS and suspended with 125 µL of PBS and 100 µL of suspension (~800,000 cells) were dispensed in a clear flat bottomed 96-well microtiter plate and the fluorescence of FITC read with a microplate fluorimeter.

### 4.6. Measurement of Total Proteins with Bicinchoninic Acid

In order to account for possible differences in the number of cells loaded in the wells, we determined the total proteins content per well and the values of fluorescence intensity throughout the paper were expressed as RFU/µg of total protein. Briefly, the cells were lysed with 1% Triton X-100 (final concentration) for 30 min at 4 °C and 25 µL of lysate or standard (Bovine Serum Albumin, BSA, in the range 0.25–1 mg/mL) were dispensed in a flat bottomed microtiter plate and the total proteins content was measured with the Bicinchoninic Acid Assay by using a commercially available kit (Sigma-Aldrich, Milan, Italy, Cat. No. BCA1), according to the manufacturer instructions. 

### 4.7. Under-Agarose Assay

Neutrophil chemotaxis was evaluated by using the under agarose assay essentially as described elsewhere [41]. Briefly, Petri dishes (35 × 10 mm) were coated with 10% FBS in PBS for 30 min at room temperature and washed twice with PBS. Then, the plates were filled with 3 mL of a 0.45% agarose dissolved in 50% HEPES-buffered complete HBSS and 50% supplemented with 20% FBS. The agarose was then allowed to solidify and two wells 3.5 mm diameter and 2.2 mm apart were cut into each gel. The gels were then equilibrated at 37 °C for 1 h and 10 µL of chemokine (1 pmol of fMLP or 10 pmol of IL-8) were loaded in the outer well, whereas 10 µL of 1 × 10^7^ cells/mL (1 × 10^5^ cells) not treated or pretreated for 15 min with 5 mg/mL of HES dissolved in complete HBSS were loaded in the inner well. After 1.5 h of incubation at 37 °C, images of migrating neutrophils were acquired every 20 s for 20 min, with a Canon EOS 700D digital reflex attached to an inverted microscope (Olympus IMT-2) equipped with a thermostatic chamber. The tracks of the migrating cells were then acquired by using ImageJ with multitracker plugin and the coordinates of the cells were analyzed by using a free software (Ibidi Chemotaxis and Migration Tool), in order to obtain quantitative parameters of migration such as FMI (forward migration index, parallel to the gradient and calculated as the ratio between the x coordinate of the cell’s end point of migration and the total distance accumulated), directionality (representing a measurement of the directness of cell trajectories, the ratio of the Euclidian distance and the total accumulated distance of a cell) and velocity (displacement/time).

### 4.8. Static Adhesion Assay on Fibrinogen and JAM-1

To evaluate the in vitro adhesion of neutrophils on substrates such as fibrinogen and JAM-1 (Junction Adhesion Molecule-1) in the absence or presence of HES, the cells were washed twice with Ca-Mg-free HBSS and then fluorescently labeled by incubation with 3 µM of calcein-AM (Sigma-Aldrich, cat. No. C1359) diluted in Ca-Mg-free HBSS for 30 min at 37 °C. The cells were centrifuged 5 min at 400× *g*, washed twice with Ca-Mg-free HBSS, and then suspended in complete HBSS and treated/not treated with 5 mg/mL HES for 15 min at 37 °C. The cells were further centrifuged 5 min at 400× *g*, washed once with complete HBSS and suspended in the same buffer at the concentration of 1 × 10^6^ cells/mL. Finally, 50 µL of cell suspension (corresponding to 50,000 cells) were dispensed in wells of a 96-well plate ELISA plate (Nunc MaxiSorp, Thermofisher Scientific, Monza, Italy; Cat. No. 44-2404-21) pre-coated with fibrinogen (Sigma-Aldrich, Milan, Italy; cat. No. F3879) or JAM-1 (AbCam, Cambridge, UK; cat. No. ab132180). Coating was performed overnight at 4 °C by dispensing 50 µL of protein prepared at the final concentration of 10 µg/mL in modified-PBS (pH 7.4, containing 1 mM CaCl_2_ and 2 mM MgCl_2_). After one washing step with modified-PBS, the wells were incubated with 100 µL of 1% BSA dissolved in modified-PBS for 1 h at room temperature. At the end of the incubation, the wells were washed one time with modified-PBS and 50 µL of labeled cell suspension were dispensed in the wells. After 30 min of incubation at 37 °C, the total fluorescence was read (excitation: 490 nm; emission 525 nm), giving the pre-wash fluorescence. The wells were then gently washed three times with modified-PBS and fluorescence per well was measured again, giving the post-wash fluorescence. The ratio between the post-wash and pre-wash fluorescence multiplied by 100 represented the percentage of adherent cells per well.

### 4.9. Western Blot Analysis of Total Phospo-Tyrosine, Akt1/2/3 and p38

The activation of integrins by HES was evaluated by Western blot analysis of total phospho-Tyrosine on cells treated with HES, ICAM-1 or not treated as a control. In addition, we evaluated specific pathways activation by determining Akt1/2/3 and p38 phosphorylation status. Briefly, 2 × 10^6^ cells were incubated in complete HBSS without stimuli, in the presence of 5 mg/ml of HES or 10 µg/mL of ICAM-1 (Sigma-Aldrich, cat. No. SRP3057) for 15 min at 37 °C. Cells were then centrifuged for 5 min at 400× *g* at 4 °C, washed once in PBS and then incubated for 30 min at 4 °C in lysis buffer containing phosphatase and protease inhibitors (50 mM Tris, pH 7.6, 150 mM NaCl, 25 mM β-Glycerophosphate, 50 mM NaF, 2 mM Na_3_VO_4_, 5 mM EDTA, 5 mM EGTA, 1% Triton X-100, Halt protease Inhibitor cocktail, Thermo Scientific, Cat. No. 87786). Total protein content was evaluated by the Bradford assay and 20 µg of proteins were loaded onto a 10% sodium dodecyl sulphate polyacrylamide gel (SDS-PAGE) under reducing conditions and electrophoresed for 45 min at 35 mA constant amperage. The separated proteins were then transferred at 300 mA for 90 min to a 0.45 µm-pore PVDF (Polyvinylidene fluoride) membrane (Immobilon-P, Merck, Darmstadt, Germany; Cat. No. IPVH00010) and the non-specific sites were blocked by incubating the membranes for 1 h at room temperature with 5% BSA (Sigma-Aldrich, Fraction V, Cat. No. 05482) in TBS-T (Tris Buffered Saline, pH 7.4, 0.1% Tween-20). After washing steps in TBS-T, the membrane was incubated overnight at 4 °C with either anti-phospho-Tyrosine (Elabscience, Huston, Texas, USA; Cat. No. E-AB-21335, diluted 1:2000), anti-beta-actin (Sigma-Aldrich, Milan, Italy; Cat. No. A3854, diluted 1:100,000), pAkt1/2/3 (1:2000), total Akt1/2/3 (1:2000), p-p38 (1:2000) or total p38 (1:2000) antibodies. Afterwards, the membrane was washed three times with TBS-T, incubated with the proper secondary antibody (diluted 1:100,000 in blocking buffer) for one hour at room temperature and washed three times each with TBS-T and TBS. Finally, the bands were revealed by ECL system (SuperSignal West Femto, Thermo Fisher Scientific, Monza, Italy; Cat. No. 34095) and film exposure. Band intensity analysis was carried out by using the program ImageStudio Lite v5.2 (Li-Cor Biosciences, Lincoln, NE, USA) and expressed as ratio relative to the loading control (beta-actin or the total form of Akt1/2/3 or p38) and normalized by the not treated condition.

### 4.10. Statistical Analysis

The comparisons of fluorescence between groups and the percentage of adherent cells on different substrates were performed by *t*-test or by 1-way ANOVA followed by Sidak’s multiple comparison test. The comparisons of the migration parameters between the HES-treated cells and the control were performed by Mann-Whitney U test. The comparisons between the normalized band intensities from western blots were performed by *t*-tests. All the analyses were carried out with GraphPad^®^ Prism 6 and a *p* < 0.05 was considered significant.

## Figures and Tables

**Table 1:** Fluorescence intensities of HES-FITC treated cells measured after quenching of the extracellular signal with trypan blue.

HES-FITC Concentration	pH 5.8	+Trypan Blue	% of Quenching	
5 mg/mL	170.9 ± 4.5	8.6 ± 0.6	95
2 mg/mL	134.3 ± 9.7	1.4 ± 0.7	99
1 mg/mL	79.0 ± 3.8	1.6 ± 0.7	98

Neutrophils were treated with trypan blue or buffer acetate at pH 5.8 and the fluorescence measured and adjusted for the total protein content in the wells. Data are expressed as mean RFU (Relative Fluorescence Units)/µg of total protein ± SD (*n* = 3).

## Supplementary Materials

Supplementary materials can be found at http://www.mdpi.com/1422-0067/20/4/817/s1.
