# Supplementary material for: Hydroxyethyl Starch 130/0.4 Binds to Neutrophils Impairing Their Chemotaxis through a Mac-1 Dependent Interaction
Source: Int J Mol Sci. 2019 Feb 14;20(4):817. doi: 10.3390/ijms20040817 (PMC6413098; doi:10.3390/ijms20040817)
Supplement: Supplementary file 1 [file ijms-20-00817-s001.pdf]

Determination of the behavior of HES from different sources

HES synthesized from different sources (potato or maize starch) was labeled by following the protocol outlined in the Material and Methods section. The cells (8 x 10^6 cells) were treated with different concentrations of HES-FITC (5 mg/ml, 2 mg/ml or 1 mg/ml) suspended in complete HBSS and incubated for 15 minutes at 37 °C. After two washing steps, cells were suspended in 1 ml of complete HBSS and 100 µl of suspension (800,000 cells) were suspended in quadruplicate in a clear flat bottomed 96-well microtiter plate and the fluorescence of FITC read with a microplate fluorimeter. The values of fluorescence were adjusted for the different labeling efficiency of HES derived from the two sources as well as for the total content of proteins. As displayed in Figure S1, we did not observe a substantial change in the behavior on integrin binding (ANOVA, $p = 0.345$) suggesting that the two kinds of HES demonstrate the same bioequivalence with respect to the binding of neutrophils.

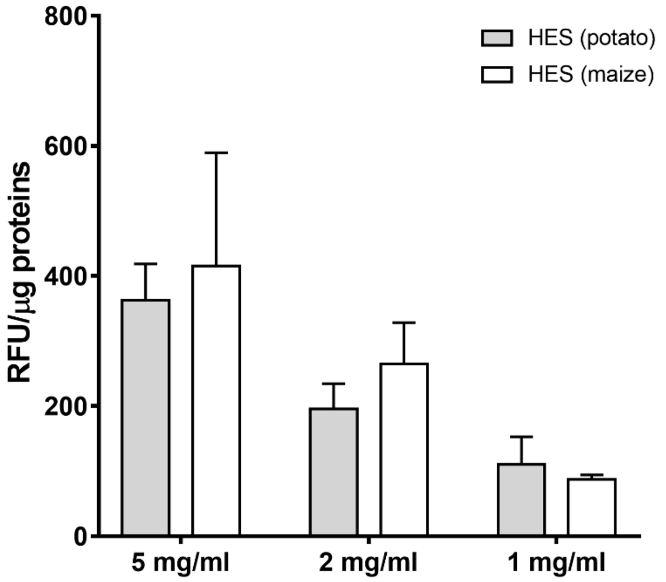

Figure S1. Evaluation of the behavior of HES derived from potato starch (grey bars) or maize starch (open bars). As outlined in the figure, we did not find any difference in the binding to the plasma membrane of neutrophils. Values represent the mean of three independent experiments.

Determination of the responsiveness of neutrophils isolated from fresh buffy coats

The cells were isolated as described in the Material and Methods section and then 200,000 cells were dispensed in different tubes according to the following scheme.

1) Not treated (control)
2) Treated with 0.1 µg/ml fMLP
3) Treated with 1 µg/ml fMLP
4) Isotypic control

After incubation at 37 °C for 5 minutes, the cells were centrifuged 5 minutes at 400 g and then incubated with anti-CD62L (diluted 1:10; Biorbyt, Cat. No. ORB156313) and anti-CD11b (diluted 1:200; Elabscience, Cat. No. E-Ab-1408) for 30 minutes at 4 °C. After three washing steps with PBS, cells were fixed for 15 minutes with 1% PFA (paraformaldehyde) in PBS and then washed one time with PBS. Finally, the cells were incubated with secondary antibodies diluted 1:100 in PBS and incubated in the dark for 30 minutes at 4 °C. At the end of the incubation, the cells were washed two times with PBS and suspended in 3% BSA + 1% NaN_3 dissolved in PBS.

The cells were analyzed by a cytofluorimeter (BD Facs Canto) and the data elaborated with the software FlowJo VX.

As displayed in Figure S2, the cells were still responsive after the isolation procedure, since the stimulation with fMLP caused an increase in CD11b and a decrease in CD62L which shed from the cell surface upon stimulation.

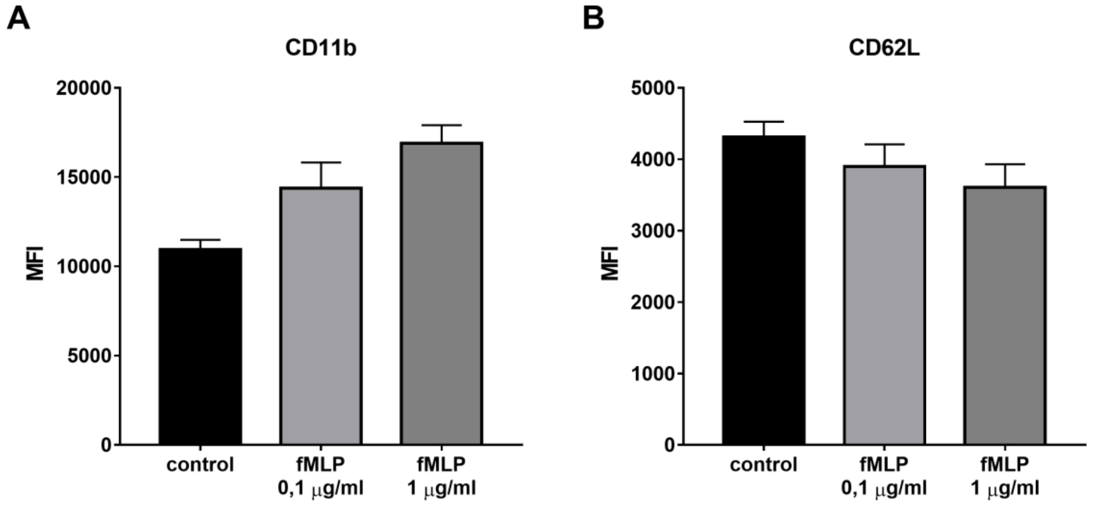

Figure S2. Evaluation of neutrophils responsiveness after isolation from fresh buffy coats. As displayed, neutrophils were responsive to stimulation by fMLP as highlighted by the increased CD11b and decreased CD62L. The data are expressed as MFI and are the mean of three independent experiments.
